# Supplementary material for: Post-Hypoglycemic hyperglycemia are highly relevant markers for stratification of glycemic variability and partial remission status of pediatric patients with new-onset type 1 diabetes
Source: PLoS One. 2023 Nov 30;18(11):e0294982. doi: 10.1371/journal.pone.0294982 (PMC10688654; doi:10.1371/journal.pone.0294982)
Supplement: Attachment — Submitted filename: PONE-D-22-33441_SND.pdf [file pone.0294982.s001.pdf]

PLOS ONE

Post-Hypoglycemic Hyperglycemia Are Highly Relevant Markers For Stratification Of Glycemic Variability and Remission Status Of Pediatric Patients With New-Onset Type 1 Diabetes.

--Manuscript Draft--

**Manuscript Number:**	PONE-D-22-33441
**Article Type:**	Research Article
**Full Title:**	Post-Hypoglycemic Hyperglycemia Are Highly Relevant Markers For Stratification Of Glycemic Variability and Remission Status Of Pediatric Patients With New-Onset Type 1 Diabetes.
**Short Title:**	GLUREDIA study
**Corresponding Author:**	Antoine Harvengt<br>Cliniques Universitaires Saint-Luc<br>Woluwe-Saint-Lambert, BELGIUM
**Keywords:**	Biomarkers, Children, Clustering, Continuous glucose monitoring, Glycemic variability, Hypoglycemia, Insulin dose-adjusted A1C, Partial Remission, Type 1 Diabetes, Therapeutic management.
**Abstract:**	Aims: To evaluate whether parameters of post-hypoglycemic hyperglycemia (PHH) correlated with glycemic homeostasis during the first year after type 1 diabetes onset and helped to distinguish pediatric patients undergoing partial remission or not.<br>Methods: In the GLUREDIA study, longitudinal values of clinical parameters, continuous glucose monitoring metrics and residual beta-cell secretion from children with new-onset type 1 diabetes were analyzed during one year. PHH parameters were calculated using an in-house built algorithm. Cross-sectional correlations between PHH parameters (i.e., PHH frequency, duration, AUC xx) and glycemic homeostasis markers were performed using adjusted mixed-effects models.<br>Results: PHH parameters were strong markers to differentiate remitters from non-remitters, the most sensitive being PHH/Hyperglycemia duration ratio. Among those, PHHAUC correlated with clinical parameters and continuous glucose monitoring metrics and inversely correlated with residual beta-cell secretion. Furthermore, combination of PHH parameters identified groups of patients that might benefit from distinct therapeutic management. Finally, patient classification into four glucotypes, as previously described, independently validated PHH parameters as reliable markers of glycemic homeostasis and improved the segregation of patients with intermediate values of IDAA1C and CPEPEST.<br>Conclusion: PHH parameters are new minimal-invasive markers of partial remission and glycemic homeostasis during the first year of type 1 diabetes that allow patient-specific therapeutic management.
**Order of Authors:**	Antoine Harvengt
	Philippe Lysy, MD - Ph-D
	Olivier Pollé
	Manon Martin
	Aline van Maanen, Ph.D.
	Laurent Gatto, Ph.D.
**Additional Information:**	
**Question**	**Response**
**Financial Disclosure**<br><br>Enter a financial disclosure statement that describes the sources of funding for the work included in this submission. Review	Grant funding from Leona M. and Harry B. Helmsley Charitable Trust

the submission guidelines for detailed requirements. View published research articles from *PLOS ONE* for specific examples.<br><br>This statement is required for submission and **will appear in the published article** if the submission is accepted. Please make sure it is accurate.<br><br>**Unfunded studies**<br>Enter: *The author(s) received no specific funding for this work.*<br><br>**Funded studies**<br>Enter a statement with the following details:<br>• Initials of the authors who received each award<br>• Grant numbers awarded to each author<br>• The full name of each funder<br>• URL of each funder website<br>• Did the sponsors or funders play any role in the study design, data collection and analysis, decision to publish, or preparation of the manuscript?<br>• **NO** - Include this sentence at the end of your statement: *The funders had no role in study design, data collection and analysis, decision to publish, or preparation of the manuscript.*<br>• **YES** - Specify the role(s) played.<br><br>* typeset	
**Competing Interests**<br><br>Use the instructions below to enter a competing interest statement for this submission. On behalf of all authors, disclose any competing interests that could be perceived to bias this work—acknowledging all financial support and any other relevant financial or non-financial competing interests.<br><br>This statement is required for submission and **will appear in the published article** if the submission is accepted. Please make sure it is accurate and that any funding sources listed in your Funding Information later in the submission form are also	The authors have declared that no competing interests exist.

declared in your Financial Disclosure statement.<br><br>View published research articles from *PLOS ONE* for specific examples.<br><br>**NO authors have competing interests**<br><br>Enter: *The authors have declared that no competing interests exist.*<br><br>**Authors with competing interests**<br><br>Enter competing interest details beginning with this statement:<br><br>*I have read the journal's policy and the authors of this manuscript have the following competing interests: [insert competing interests here]*<br><br>* typeset	
**Ethics Statement**<br><br>Enter an ethics statement for this submission. This statement is required if the study involved:<br><br>• Human participants<br>• Human specimens or tissue<br>• Vertebrate animals or cephalopods<br>• Vertebrate embryos or tissues<br>• Field research<br><br>Write "N/A" if the submission does not require an ethics statement.<br><br>General guidance is provided below. Consult the submission guidelines for detailed instructions. **Make sure that all information entered here is included in the Methods section of the manuscript.**	Comité d'Ethique Hospitalo-Facultaire of CUSL (2018/04DEC/462) and is registered in www.clinicaltrial.gov (NCT04007809)<br><br>Written

Powered by Editorial Manager® and ProduXion Manager® from Aries Systems Corporation

**Format for specific study types**<br><br>**Human Subject Research (involving human participants and/or tissue)**<br>• Give the name of the institutional review board or ethics committee that approved the study<br>• Include the approval number and/or a statement indicating approval of this research<br>• Indicate the form of consent obtained (written/oral) or the reason that consent was not obtained (e.g. the data were analyzed anonymously)<br><br>**Animal Research (involving vertebrate animals, embryos or tissues)**<br>• Provide the name of the Institutional Animal Care and Use Committee (IACUC) or other relevant ethics board that reviewed the study protocol, and indicate whether they approved this research or granted a formal waiver of ethical approval<br>• Include an approval number if one was obtained<br>• If the study involved *non-human primates*, add *additional details* about animal welfare and steps taken to ameliorate suffering<br>• If anesthesia, euthanasia, or any kind of animal sacrifice is part of the study, include briefly which substances and/or methods were applied<br><br>**Field Research**<br><br>Include the following details if this study involves the collection of plant, animal, or other materials from a natural setting:<br>• Field permit number<br>• Name of the institution or relevant body that granted permission	
**Data Availability**<br><br>Authors are required to make all data underlying the findings described fully available, without restriction, and from the time of publication. PLOS allows rare exceptions to address legal and ethical concerns. See the PLOS Data Policy and FAQ for detailed information.	Yes - all data are fully available without restriction

A Data Availability Statement describing where the data can be found is required at submission. Your answers to this question constitute the Data Availability Statement and **will be published in the article**, if accepted.<br><br>**Important:** Stating 'data available on request from the author' is not sufficient. If your data are only available upon request, select 'No' for the first question and explain your exceptional situation in the text box.<br><br>Do the authors confirm that all data underlying the findings described in their manuscript are fully available without restriction?	
**Describe where the data may be found in full sentences. If you are copying our sample text, replace any instances of XXX with the appropriate details.**<br><br>• If the data are **held or will be held in a public repository**, include URLs, accession numbers or DOIs. If this information will only be available after acceptance, indicate this by ticking the box below. For example: *All XXX files are available from the XXX database (accession number(s) XXX, XXX.).*<br>• If the data are all contained **within the manuscript and/or Supporting Information files**, enter the following: *All relevant data are within the manuscript and its Supporting Information files.*<br>• If neither of these applies but you are able to provide **details of access elsewhere**, with or without limitations, please do so. For example:<br><br>*Data cannot be shared publicly because of [XXX]. Data are available from the XXX Institutional Data Access / Ethics Committee (contact via XXX) for researchers who meet the criteria for access to confidential data.*<br><br>*The data underlying the results presented in the study are available from (include the name of the third party*	All relevant data are within the manuscript and its Supporting Information files

Powered by Editorial Manager® and ProduXion Manager® from Aries Systems Corporation

and contact information or URL).<br>• This text is appropriate if the data are owned by a third party and authors do not have permission to share the data.<br><br>* typeset	
Additional data availability information:	Tick here if the URLs/accession numbers/DOIs will be available only after acceptance of the manuscript for publication so that we can ensure their inclusion before publication.

Powered by Editorial Manager® and ProduXion Manager® from Aries Systems Corporation

Abstract + Article
Click here to access/download;Manuscript;GLUREDIA Abstract + article.pdf

Original article

Post-Hypoglycemic Hyperglycemia Are Highly Relevant Markers For Stratification Of Glycemic Variability and Remission Status Of Pediatric Patients With New-Onset Type 1 Diabetes.

Antoine A. Harvengt [1,2]*, Olivier G. Polle [1,2]*, Manon Martin [3], Aline van Maanen Ph.D. [4], Laurent Gatto Ph.D. [3] and Philippe A. Lysy Ph.D. [1,2].

*These authors equally contributed to this work.

[1]Pôle PEDI, Institut de Recherche Expérimentale et Clinique, UCLouvain, Brussels, Belgium

[2]Specialized Pediatrics Service, Cliniques universitaires Saint-Luc, Brussels, Belgium

[3]Computational Biology and Bioinformatics (CBIO) Unit, de Duve Institute, UCLouvain, Brussels, Belgium

[4]Statistical Support Unit, Institut Roi Albert II, Cliniques universitaires Saint-Luc, Brussels, Belgium

Corresponding Author:

Full name: **Philippe A. Lysy.**

Department: **Specialized Pediatrics Service.**

Institute/University/Hospital: **Cliniques universitaires Saint-Luc.**

Street Name & Number: **Avenue Hippocrate, 10.**

City, State, Postal code, Country: **1200, Brussels, Belgium.**

Tel: **003227641370.**

Fax: 003227648991.

E-mail: **philippe.lysy@uclouvain.be.**

Word count: **3893 words**.

Key words: Biomarkers, Children, Clustering, Continuous glucose monitoring, Glycemic variability, Hypoglycemia, Insulin dose-adjusted A1C, Partial Remission, Type 1 Diabetes, Therapeutic management.

ABSTRACT

Aims: To evaluate whether parameters of post-hypoglycemic hyperglycemia (PHH) correlated with glycemic homeostasis during the first year after type 1 diabetes onset and helped to distinguish pediatric patients undergoing partial remission or not.

Methods: In the GLUREDIA study, longitudinal values of clinical parameters, continuous glucose monitoring metrics and residual beta-cell secretion from children with new-onset type 1 diabetes were analyzed during one year. PHH parameters were calculated using an in-house built algorithm. Cross-sectional correlations between PHH parameters (i.e., PHH frequency, duration, AUC xx) and glycemic homeostasis markers were performed using adjusted mixed-effects models.

Results: PHH parameters were strong markers to differentiate remitters from non-remitters, the most sensitive being PHH/Hyperglycemia duration ratio. Among those, PHH_{AUC} correlated with clinical parameters and continuous glucose monitoring metrics and inversely correlated with residual beta-cell secretion. Furthermore, combination of PHH parameters identified groups of patients that might benefit from distinct therapeutic management. Finally, patient classification into four glucotypes, as previously described, independently validated PHH parameters as reliable markers of glycemic homeostasis and improved the segregation of patients with intermediate values of $IDAA_{1C}$ and $CPEP_{EST}$.

Conclusion: PHH parameters are new minimal-invasive markers of partial remission and glycemic homeostasis during the first year of type 1 diabetes that allow patient-specific therapeutic management.

INTRODUCTION

Type 1 diabetes mellitus is characterized by a progressive decline in beta-cell mass, resulting in a clinical state of insulinopenia when beta-cell function drops below twenty percent [1], which defines the clinical disease onset. From that moment, patients with type 1 diabetes rely on a combination of exogenous insulin administration, healthy diet and regular physical activity to achieve optimal glycemic control [2-4]. Immediately after disease onset and insulin therapy initiation, the majority of patients ('occurrence rate: [40–75%]) experience a period of partial remission defined by the coexistence of low levels of glycemic variability and reduced exogenous insulin requirements [5]. However, after partial remission, a progressively growing dependence on exogenous insulin induces an increased glycemic variability and the difficulty for patients to avoid hypoglycemia. Indeed, despite major improvements in diabetes management [6], nearly half of type 1 diabetes patients do not reach recommended therapeutic targets [7]. Also, under classical intensive insulin therapy, an incompressible hypoglycemia frequency (estimated at 5-15% of total glucose values [8]) is unavoidable to maintain mean glycemia within targets.

Hypoglycemia is the most common complication in type 1 diabetes patients [8] and corresponds to an inadequacy between insulin substitution, insulin needs and carbohydrate intake with a consecutive drop of glycemia (i.e., below 60 mg/dL) [9]. Hypoglycemic events are either asymptomatic or associated with mild-to-severe clinical manifestations, such as convulsions and/or loss of consciousness [10,11]. Though rarely life-threatening [8], children experience on average three symptomatic hypoglycemic events per week that commonly require external intervention (i.e., providing oral carbohydrates or glucagon analogs) [8]. Additionally, as hypoglycemia is an acute, stressful and unpleasant event for both the child and the parents, these

events may lead to excessive carbohydrate intake followed by acute hyperglycemia and, more globally, increased glycemic variability [12]. This highlights hypoglycemia as a potential trigger of hyperglycemic excursions and the need for therapeutic education focusing on individual profiles of glycemic variation.

In patients with type 1 diabetes, hypoglycemic events were associated with increased oxidative stress [13-15] that participates in the development of microvascular complications (e.g., diabetic retinopathy and nephropathy) [16,17]. This was clinically demonstrated by Ceriello et al. who observed increased expression of oxidative stress markers in patients experiencing hypoglycemia followed by hyperglycemia, while the same markers remained unchanged when a normoglycemic state was maintained [18]. These results suggest a link between inadequate carbohydrate intake during hypoglycemia and the development of diabetes complications.

Based on these observations, our team recently introduced the concept of post-hypoglycemic hyperglycemia (PHH) and investigated their influence on diabetes control (EPHICA study) [19]. PHH was defined as a hypoglycemia (i.e., glycemia <60 mg/dL) followed, within a 2-hour period, by hyperglycemia (i.e., glycemia >160 mg/dL) **(Fig. 1).** In pediatric patients with longstanding type 1 diabetes (i.e., clinical onset >1 year), these glycemic patterns represented more than a third of time spent in hyperglycemia for nearly 15% of patients and displayed strong correlations with markers of glycemic variability [19]. While current beta-cell function markers lack reliability in reflecting the extents of glycemic homeostasis in patients with type 1 diabetes, our recent results support PHH as reliable and minimal-invasive markers of glycemic homeostasis [20] that may help individualizing therapeutic interventions.

The objectives of our GLUREDIA study were to characterize PHH events in a cohort of pediatric patients with new-onset type 1 diabetes and investigate whether

PHH parameters correlated with glycemic homeostasis including clinical parameters (e.g., HbA_{1C}, total daily dose of insulin, Insulin-Dose Adjusted A1C), continuous glucose monitoring metrics or residual beta-cell secretion (i.e., $CPEP_{EST}$). Also, we evaluated whether these PHH parameters supported the clinical heterogeneity suggested by the newly identified glucotypes [21] during the first year after T1D onset.

METHODOLOGY

Study design & participants

GLUREDIA is a subsidiary study of the multicentric DIATAG study that was previously described [21]. Briefly, DIATAG included new-onset pediatric patients with type 1 diabetes aged between 6 months and 17 years old. ~~type~~ 1 diabetes was diagnosed using International Society for Pediatric and Adolescent Diabetes (ISPAD) criteria [1] and patients were positive for at least one anti-islet autoantibody (i.e., anti-insulin, anti-protein tyrosine phosphatase, anti-glutamic acid decarboxylase, anti-Zinc transporter 8). All participants and their parents gave their written consent prior to enrolment in the study. The protocol was approved by the seven participating ethical committees (Comité d'Ethique Hospitalo-Facultaire of CUSL (2018/04DEC/462) and is registered in www.clinicaltrial.gov (NCT04007809). Exclusion criteria are described elsewhere [21]. All participants were without significant comorbidities at inclusion (i.e., high blood pressure (>P95), proteinuria (>0.15 g/L) or body mass index [BMI] Z-score >+3 DS according to Cole et *al*. [22]~~)~~.

A data array was collected at diagnosis and included demographics of the patient (i.e., age at diagnosis, sex, pubertal status, weight, height and BMI) and diabetes characteristics (i.e., presence of ketoacidosis at diagnosis, anti-islet antibodies, insulin regimen). From diagnosis, clinical parameters (i.e., Insulin-Dose Adjusted. A1C [$IDAA_{1C}$], HbA_{1C}, total insulin daily dose [TDD]) and raw continuous glucose monitoring metrics were collected ~~at~~ each outpatient clinical visit (i.e., every 3 months) for 1 year. All data were ~~gathered inside~~ the Research Electronic Data Capture (REDCap) system provided by Vanderbilt University (Nashville, USA) and hosted at Cliniques universitaires Saint-Luc.

Continuous glucose monitoring metrics analysis and PHH detection

Raw continuous glucose monitoring metrics from a 90-day interval were extracted at each outpatient clinical visit from various continuous glucose monitoring devices (i.e., Freestyle Libre®, Abbott; Dexcom®, Dexcom; EnliteTM, Medtronic MiniMed). Raw glycemic data were pre-processed using the R (R Core Team (2021)) statistical package cgmanalysis. Data quality check and calculation of a panel of forty-six continuous glucose monitoring metrics were performed using the Iglu statistical package.

For PHH detection, raw continuous glucose monitoring metrics were aggregated on T-SQL™ (Azure SQL Instance) and, quality-checked and optimized using in-house PHH detection algorithm implemented on C# 7.3 using .NET framework. continuous glucose monitoring metrics exhibiting a time lapse above 45 minutes between consecutive glycemic values were excluded from the analysis. Glycemic values below 20 mg/dL or displaying a glycemic change above 100 mg/dL in less than 5 minutes were considered as artifacts and further excluded from the analysis. Manual inspection of PHH patterns for algorithm accuracy-check was performed using PowerBI (Microsoft^{TM}). PHH event was defined as a hypoglycemic episode (i.e., interstitial glucose <60 mg/dL) followed, within two hours, by a hyperglycemic episode (i.e., interstitial glucose >160 mg/dL). The PHH event starts when a value above 160 mg/dL is detected within two hours after hypoglycemia. If a glycemic value below 160 mg/dL (e.g., 155 mg/dL) is identified during the PHH event and followed, within a maximum of 15 minutes, by a glycemic value above 160 mg/dL; the PHH event continues. The ends when two consecutive glycemic values below 160 mg/dL (i.e., >15 minutes) are detected. The PHH duration corresponds to the difference in minutes between the start and the end of PHH. The area under the curve (AUC) of PHH was calculated using the

irregular polygon rule [23] where PHH starts and ends are defined as y=0. The algorithm will be added to GitHub repository.

Partial remission

Partial remission was defined by $IDAA_{1C} = HbA_{1C} + (4 \times \text{insulin doses/kg/d})$ [24], where a score below 9 defines remitters and a score above 9 defines non-remitters.

Residual C-peptide secretion

Residual C-peptide secretion ($CPEP_{EST}$) was evaluated at +3 and +12 months after diagnosis. Stimulated C-peptide values were estimated using a mathematical formula described by Wentworth *et al* ($\text{Log}_e\ [CPEP_{EST} + 1] = 0.317 + 0.00956 \times BMI - 0.000159 \times \text{duration} + 0.710 \times FCP - 0.0117 \times FPG - 0.0186 \times HbA_{1C} - 0.0665 \times \text{insulin}$, where BMI is in kg/m^2, duration is in days, Fasting C-Peptide [FCP] is in nmol/l, Fasting plasma glucose [FPG] is in mmol/L, HbA_{1C} is in % and insulin is in IU/kg) [25]. Fasting C-peptide and plasma glucose values were determined at the central laboratory of CUSL for all samples. C-peptide was measured using a two-site chemiluminescence immunoassay (LiaisonXL®, Diasorin, France).

Glucotypes

Patient glucotypes were recently described by our clinical research center using hierarchical clustering based on continuous glucose monitoring metrics and clinical parameters [21]. Briefly, these glucotypes exhibited specific 24 hours profiles of continuous glucose monitoring metrics and refined the current dichotomic definition of partial remission.

Statistical analyses

Nearly all statistical analyses were performed R (R Core Team [2021]. R: A language and environment for statistical computing. R Foundation for Statistical Computing, Vienna, Austria. URL [https://www.R-project.org/]). In the end, Roc analyses were done using SAS v.9.4 ([https://www.sas.com/en_us/home.html]). The statistical significance level used for all analyses was 0.05. PHH parameters were transformed using Box-Cox transformation when needed. Demographic and clinical data are reported as mean ± SD for continuous variables and as numbers and proportions for categorical variables. Comparisons between groups were performed using Student's t test, chi-square test and linear regression or their nonparametric equivalent (Mann–Whitney U test and Kruskal–Wallis test, respectively) as appropriate. P-values were adjusted for multiple testing with the Bonferroni procedure [26]. Marginal R^2 (coefficient of determination) between PHH parameters and secretion, continuous glucose monitoring metrics and clinical parameters were calculated using generalized linear mixed models [27] with R packages lme4 [28] and lmerTest [29] to take into account multiple measurements from the same patient. Models included the methods as fixed effects and patient identity as a random intercept. Residuals were inspected for normality on Q-Q plots.

RESULTS

Clinical and anthropometric characteristics of the GLUREDIA cohort

Seventy-one pediatric patients with new-onset type 1 diabetes were quarterly followed during the first year after diagnosis, corresponding to a total of 241 outpatient visits. Of these visits, 48 were excluded as they did not fulfill our pre-established quality criteria or had missing associated clinical data. Final analysis was performed on 193 clinical visits from 66 patients (i.e., 59 patients at +3, 48 patients at +6, 45 patients at +9 and 41 patients at 12 months after the diagnosis) representing a total of 1900000 interstitial glucose values. The baseline characteristics (i.e., at diagnosis) of the cohort are described in **Table 1**.

PHH are frequent during the first year after type 1 diabetes onset

PHH parameters were calculated at each time point from a 3-month continuous glucose monitoring metrics period and included the proportion of total duration of all PHH according to total time spent in hyperglycemia (PHH/Hyperglycemia duration ratio) and the hypoglycemia proportion followed by a PHH (PHH/Hypoglycemia frequency ratio). Of the 193 analyzed continuous glucose monitoring metrics, 174 (90%) exhibited at least one PHH event in the 3-month period. Globally, participants presented 0.19 (±0.20) PHH events/day that lasted on average 155 (±117) minutes corresponding to a mean PHH_{AUC} of 32023 (±30037). The PHH/Hyperglycemia duration ratio was 0.04 (±0.05) and the PHH/Hyperglycemia frequency ratio was 0.26 (±0.18), whereas the PHH/Hypoglycemia frequency ratio was 0.35 (±1.06) meaning that, on average, about a third of hypoglycemia were followed by hyperglycemia in our patient cohort **(Table 1).**

Next, we investigated the circadian rhythm of the PHH, as physiological phenomena or behavioral aspects might influence the occurrence of these events (e.g., Somogyi effect [30], exogenous carbohydrate intake). We subdivided the whole day into four distinct periods: morning (5 a.m.; 10 a.m.; morning$_{5-10}$), day (10 a.m.; 4 p.m.; day$_{10-4}$), evening (4 p.m.; 10 p.m.; evening$_{4-10}$) and night (10 p.m.; 5 a.m.; night$_{10-5}$). We observed a higher PHH frequency during the day$_{10-4}$ and evening$_{4-10}$ than in the morning$_{5-10}$ and night$_{10-5}$ ($p<0.001$) (**Table 1**). On the other hand, the PHH$_{AUC}$ increased progressively according to the different daytime periods: it was the highest at night$_{10-5}$ ($p<0.05$).

PHH are thus a common phenomenon in patients with *de novo* type 1 diabetes and demonstrate high inter-patient variability (i.e., high SD) for each global parameter. Evaluation of PHH across the day revealed that these occurred more frequently during the day$_{10-4}$ while being of longer duration during the night$_{4-10}$.

Partial remission is associated with fewer and lower PHH occurrence

We next investigated whether PHH parameters reflected the occurrence and intensity of partial remission in patients with new-onset type 1 diabetes. Among the 193 glucose records studied, 108 (56%) belonged to patients undergoing partial remission.

Remitters presented fewer PHH events ($p<0.001$) and these were of shorter duration ($p<0.001$) and with smaller AUC ($p<0.001$) than the one observed in non-remitters. Also, remitters exhibited fewer hypoglycemia followed by a PHH (PHH/Hypoglycemia frequency ratio; $p<0.001$) concurring to a four time decrease in the percentage of time spent in hyperglycemia due to a PHH (PHH/Hyperglycemia duration ratio; $p<0.001$), as compared to non-remitters **(Table 1)**. While the biggest PHH$_{AUC}$ were observed during the night$_{10-5}$ for both groups ($p<0.001$); remitters exhibited smaller PHH$_{AUC}$ compared to non-remitters, regardless of the daytime

periods. Paradoxically, there was no significant difference in PHH frequency in the morning$_{5\text{-}10}$ and during the night$_{10\text{-}5}$ between remitters and non-remitters ($p>0.05$), contrasting with the two other daytime periods (day$_{10\text{-}4}$ and evening$_{4\text{-}10}$) where PHH frequency was higher for non-remitters than for remitters ($p<0.05$).

Supporting the clinical utility of these measures, we generated ROC curves and calculated the threshold for each parameter that distinguished remitters from non-remitters. All studied PHH parameters were able to predict the remission status of a given patient (all p-values<0.05) with PHH/Hyperglycemia duration ratio being the most sensitive parameter (ratio<0.02; Se=86% and Sp=68%) and PHH duration mean the most specific parameter (duration mean <132 min; Se=76% and Sp=74%).

Finally, investigating the correlations between the partial remission intensity (i.e., IDAA$_{1C}$ score) and PHH parameters, we found moderate and strong correlations respectively between IDAA$_{1C}$ and PHH frequency ($R^2=0.10$; $p<0.001$) or PHH$_{AUC}$ ($R^2=0.32$; $p<0.001$) **(Table 2).** Notably, large variability in the PHH frequency was observed for intermediate IDAA$_{1C}$ values (i.e., in 8-10 range)**.**

High residual C-peptide secretion is associated with a reduction of PHH parameters

As PHH parameters overlapped for intermediate IDAA$_{1C}$ scores, we evaluated whether replacing this score by a residual beta-cell secretion marker (i.e., CPEP$_{EST}$) might improve differences in PHH parameters among both remission groups. The patients were classified into four groups according to their residual estimated C-peptide secretion [31]: high (c-peptide>0.4 pmol/mL), intermediate (0.2 pmol/mL<c-peptide⩽0.4 pmol/mL), low (0.17 pmol/mL<c-peptide⩽0.2 pmol/mL), and undetectable (c-peptide⩽0.17 pmol/mL).

CPEP$_{EST}$ moderately correlated with PHH frequency (R^2=0.08; p<0.001), PHH$_{AUC}$ (R^2=0.17; p<0.001) and strongly with PHH/Hyperglycemia duration ratio (R^2=-0.29; p<0.001). In addition, no correlation was found between residual CPEP$_{EST}$ secretion and PHH/Hypoglycemia frequency ratio (R^2=0.03; p=0.13). Interestingly, patients with high residual secretion exhibited fewer and shorter PHH events, contrasting with patients with low or undetectable residual secretion (p<0.01) **(Table 2).** Finally, we observed that patients with intermediate secretion tended to have high intra-group variability in PHH frequency.

PHH parameters correlate with glycemic homeostasis markers

As correlations between PHH parameters and beta-cell residual secretion were moderate, we investigated whether PHH parameters would better correlate with continuous glucose monitoring metrics including glycemic variability (i.e., coefficient of variation [CV], mean of daily differences [MODD]) and time spent within different glycemic ranges (i.e., Time in range [TIR$_{70-180}$, 70-180mg/dL], Time above the range [TAR$_{>180}$, >180mg/dL], hypoglycemia frequency [<60mg/dL]) **(Table 2).**

CV moderately correlated with nearly all PHH parameters (p<0.05) except for PHH/hypoglycemia frequency ratio (p>0.05) (**Table 2**). Interestingly, patients with CV values >36% exhibited three times more PHH events than patients with CV <36%. Also, MODD showed the strongest correlation among all glycemic homeostasis markers with all PHH parameters (R^2>0.17), except for PHH frequency. TIR$_{70-180}$ inversely correlated with PHH parameters. Interesting, TAR$_{>180}$ weakly correlated with PHH frequency. Also, the hypoglycemia frequency best correlated with PHH frequency (R^2=0.19; p<0.001) **(Figure 2).**

Focusing on hypoglycemia and glycemic variability, patients were categorized into three different groups that demonstrated specific combinations of glycemic variability (i.e., CV) and PHH parameters (i.e., PHH_{AUC} and PHH/Hypoglycemia frequency ratio) (**Figure 3**). Group 1 corresponds to patients with stable type 1 diabetes (i.e., CV <36%) with small PHH/Hypoglycemia frequency ratio (<0.25) and very low PHH_{AUC} (<25000). Conversely, patients in Group 2 demonstrate highly variable PHH/Hypoglycemia frequency ratio (0.1-0.5) with low-to-intermediate PHH_{AUC} (<60000) and high glycemic variability (CV>36%). Finally, patients in Group 3 have very rare hypoglycemia that is usually followed by a PHH (PHH/Hypoglycemia frequency ratio [>0.4]) of high amplitude (PHH_{AUC} [>60000]). Interestingly, group 1 was mostly composed of remitters, and groups 2 and 3 from non-remitters. Finally, the temporal evolution of each patient revealed that more than half of the patients stayed in the same group (53%) during the first year of type 1 diabetes while others switched from one group to another except from Groups 2 or 3 to Group 1.

Patient glucotypes identify patients at risk for PHH.

To better characterize patients with non-discriminative indexes of glycemic homeostasis markers (i.e., intermediate $CPEP_{EST}$ secretions and $IDAA_{1C}$ scores), we studied PHH parameter distribution in the four distinct glucotypes that were previously described by our team in patients during the first year after type 1 diabetes onset [21].

Glucotypes 1 and 2 demonstrated very low PHH duration mean representing a low PHH/Hyperglycemia duration ratio (< 0.01). While PHH_{AUC} of glucotype 3 remained similar to glucotype 2, we observed a major increase in PHH frequency and a concomitant rise in PHH/Hyperglycemia duration ratio in glucotype 3. Finally, contrasting with other glucotypes, glucotype 4 demonstrated the highest PHH/Hyperglycemia duration ratio (0.1 ± 0.08). Notably, glucotypes 2 and 4 were

characterized by high PHH/Hypoglycemia frequency ratio though high variability could be observed between the patients (i.e., respectively 0.61 ± 0.23 and 0.77 ± 0.38) **(Figure 4)**.

Circadian PHH analysis showed an increase of PHH frequency in glucotypes 3 and 4 with highest differences being observed during the day$_{10-4}$ and evening$_{4-10}$ ($p<0.05$). Interestingly, PHH frequency in the morning$_{5-10}$ did not differ across glucotypes ($p>0.05$). Moreover, largest PHH$_{AUC}$ were observed during the night$_{10-4}$ ($p<0.001$) regardless of glucotypes with patients in glucotype 1 experiencing the smallest PHH$_{AUC}$.

Discussion

Partial remission reflects a transient recovery of beta-cell function with increased insulin secretion [32] and improved peripheral insulin sensitivity [33], leading to decreased dependence on exogenous insulin and optimal glycemic control (e.g., TIR_{70-180}, glycemia variability) [1]. When beta-cell function further declines, insulin requirements and glycemic variability increase, corresponding to the end of partial remission and an increased hypoglycemic risk. Current definitions and biomarkers used to identify partial remission (i.e., residual secretion [31,34] or $IDAA_{1C}$ score [24]) either require invasive blood sampling or present several limitations to describe the evolution of glycemic homeostasis (e.g., hypoglycemia, glycemic variability, insulin sensitivity) [20]. In this context, there is a need for minimal-invasive reliable markers that allow the characterization and stratification of patients with type 1 diabetes based on their glycemic status.

In our GLUREDIA study, we extensively characterized PHH, a new marker of glycemic variability, in new-onset type 1 diabetes patients. Using 12-week continuous glucose monitoring metrics, we identified PHH parameters (e.g., PHH duration mean and PHH/hyperglycemia duration ratio) as both highly sensitive and specific markers to differentiate between patients undergoing remission or not. Furthermore, we established clinically-relevant segregation thresholds for each of them. Also, most PHH parameters demonstrated high variability across the day being the highest during the day_{10-4} and $evening_{4-10}$. This suggested that reduced residual beta-cell function in combination with behavioral habits might be major triggers of PHH events. Finally, PHH parameters exhibited strong correlations with continuous glucose monitoring metrics (i.e., TIR_{70-180}, $TAR_{>180}$, CV) while weakly-to-moderately correlating with residual beta-cell secretion.

Integrating previously described glucotypes [21], we showed that PHH parameters mirrored the progressive increase of glycemia variability across the various glucotypes (**Figure 4**). Interestingly, refining the ROC curves analysis, most false positive and negative patients had an $IDAA_{1C}$ score in the 8-10 range though distinctively distributing across glucotypes 2 and 3 that were previously described by our team [21]. This observation independently validates our previous results, further confirms PHH parameters as reliable markers of glycemic homeostasis and supports recent evidence that partial remission should be considered as a continuum rather than a dichotomic phenomenon [21].

As previously mentioned, glycemic variability and hyperglycemia are both independent predictors of micro and macro-vascular complications through protein glycation (AGE) and oxidative stress [35,36]. Paradoxically, continuous glucose monitoring metrics are not integrated into the main scores defining partial remission. In our study, most PHH parameters demonstrated to be strong markers of continuous glucose monitoring metrics (e.g., CV and MODD) (**Figure 2/Table 2**). Going further, our results support the clinical validity of the diabetes stability definition based on CV [37] as a steeper increase of PHH parameters values was observed from the value of 36% [37]. Moreover, Cerriello et al. showed that PHH occurrence was an independent risk factor of oxidative stress. Indeed, patients with type 1 diabetes presenting PHH displayed a pejoration of endothelial function, an increase in inflammation and an increase of oxidative stress markers in an ischemic-reperfusion-like effect [18]. These findings together reinforce PHH parameters as reliable and independent markers of glycemic homeostasis and a potential predictor of type 1 diabetes-related chronic complications.

Carbohydrate intake and management of hypoglycemia influence glycemic control in a patient-dependent way [38]. Indeed, in our study, PHH principally occurred during the day_{10-4} and $evening_{4-10}$ when the parents and/or the child are active and may strongly influence the glycemic control by some avoidable. Following this idea, the combination of PHH parameters (PHH_{AUC} and PHH/Hypoglycemia frequency ratio) and hypoglycemia frequency subdivided our type 1 diabetes patient cohort into three subgroups. Among these groups, the majority of patients in Group 2 frequently overtreated their hypoglycemia when trying to reach a normoglycemic state resulting in short and frequent PHH, and consequently high glycemic variability. Therefore, therapeutic education focusing on the management of hypoglycemia [8] and targeting a PHH/Hypoglycemia frequency ratio below 0.25 may considerably improve disease control in these patients, by preventing further PHH events with reduced glycemic variability.

CONCLUSION

Our study deepened the characterization of PHH events during the first year of type 1 diabetes, which revealed to be recurrent in our pediatric cohort, especially when diabetes was poorly controlled. PHH parameters (e.g., PHH_{AUC} and PHH/hyperglycemia duration ratio) allowed sensitive and specific distinction between remitters and non-remitters using a minimally invasive method. Also, PHH parameters independently supported partial remission as a continuum of dysglycemia, with a longitudinal increase along the four described glucotypes. Moreover, PHH_{AUC} and PHH/hyperglycemia duration ratio demonstrated to be reliable markers of glycemic homeostasis showing moderate-to-strong correlations with most glycemic control parameters (i.e., TIR_{70-180}, $TAR_{>180}$, CV) though only weak-to-moderate correlations were found with residual beta-cell secretion. Finally, the circadian analysis of PHH events highlighted the role of exogenous carbohydrate intakes in glycemic variability where the combination of PHH/hypoglycemia frequency ratio and PHH_{AUC} segregated patients into three groups that might foster patient-specific interventions. We believe that integrating PHH parameters in continuous glucose monitoring reports will raise awareness of the hypoglycemia overtreatment and provide an additional tool for physicians to improve the individualization of their educational interventions.

The principal strength of our multicentric and pediatric study relies on the cross-sectional data analysis that integrates complementary markers of glycemic homeostasis during the first year of type 1 diabetes. In addition, the establishment of PHH parameters thresholds allows an easy, reliable and poorly-invasive determination of the remission status and assessment diabetes control using freely-available algorithm.

Our study was also limited by the cross-sectional analysis of all three parameters (i.e., clinic, secretion and continuous glucose monitoring metrics) that were only available for a subset of patients (i.e., 70%). Also, the sensor manufacturer may influence the data though most of our dataset (i.e., >90%) obtained from Freestyle Libre®. Furthermore, the small numbers of patients using pump insulin delivery system did not allowed us to perform subanalysis according to the insulin regimen (i.e., multiple daily injections vs pump). Finally, data including the type of rapid insulin analog were not collected and thus could not be analyzed.

REFERENCES

(1) Couper, J. J., Haller, M. J., Greenbaum, C. J., Ziegler, A. G., Wherrett, D. K., Knip, M., & Craig, M. E. (2018). ISPAD Clinical Practice Consensus Guidelines 2018: Stages of type 1 diabetes in children and adolescents. *Pediatric diabetes*, *19*, 20-27.

(2) DiMeglio, L. A., Acerini, C. L., Codner, E., Craig, M. E., Hofer, S. E., Pillay, K., & Maahs, D. M. (2018). ISPAD Clinical Practice Consensus Guidelines 2018: Glycemic control targets and glucose monitoring for children, adolescents, and young adults with diabetes.

(3) Danne, T., Phillip, M., Buckingham, B. A., Jarosz-Chobot, P., Saboo, B., Urakami, T., ... & Codner, E. (2018). ISPAD Clinical Practice Consensus Guidelines 2018: Insulin treatment in children and adolescents with diabetes. *Pediatric diabetes*, *19*, 115-135.

(4) Smart, C. E., Annan, F., Bruno, L. P., Higgins, L. A., & Acerini, C. L. (2014). Nutritional management in children and adolescents with diabetes. *Pediatric diabetes*, *15*(S20), 135-153.

(5) Zhong, T., Tang, R., Gong, S., Li, J., Li, X., & Zhou, Z. (2020). The remission phase in type 1 diabetes: Changing epidemiology, definitions, and emerging immuno-metabolic mechanisms. *Diabetes/metabolism research and reviews*, *36*(2), e3207.

(6) Prahalad, P., Tanenbaum, M., Hood, K., & Maahs, D. M. (2018). Diabetes technology: improving care, improving patient-reported outcomes and preventing complications in young people with Type 1 diabetes. *Diabetic Medicine*, *35*(4), 419-429.

(7) Maiorino, M. I., Signoriello, S., Maio, A., Chiodini, P., Bellastella, G., Scappaticcio, L., ... & Esposito, K. (2020). Effects of continuous glucose monitoring on metrics of glycemic control in diabetes: a systematic review with meta-analysis of randomized controlled trials. *Diabetes Care*, *43*(5), 1146-1156.

(8) Abraham, M. B., Jones, T. W., Naranjo, D., Karges, B., Oduwole, A., Tauschmann, M., & Maahs, D. M. (2018). ISPAD Clinical Practice Consensus Guidelines 2018: Assessment and management of hypoglycemia in children and adolescents with diabetes. *Pediatric diabetes*, *19*, 178-192.

(9) Young, Jacques. et Collège des enseignants d'endocrinologie, diabète et maladies métaboliques (France)., *Endocrinologie, diabétologie et maladies métaboliques*, Issy-les-Moulineaux, Elsevier-Masson, dl 2016, 589 p. Page 212 premier paragraphe.

(10) Lavens, A., Nobels, F., De Block, C., Oriot, P., Verhaegen, A., Chao, S., ... & Belgian Group of Experts IQED and IQECAD. (2021). Effect of an integrated, multidisciplinary nationwide approach to type 1 diabetes care on metabolic outcomes: an observational real-world study. *Diabetes technology & therapeutics*, *23*(8), 565-576.

(11) Maahs, D. M., Hermann, J. M., DuBose, S. N., Miller, K. M., Heidtmann, B., DiMeglio, L. A., Rami-Merhar, B., Beck, R. W., Schober, E., Tamborlane, W. V., Kapellen, T. M., & Holl, R. W. (2014). Contrasting the clinical care and outcomes of 2,622 children with type 1 diabetes less than 6 years of age in the

United States T1D Exchange and German/Austrian DPV registries. *Diabetologia*, *57*(8), 1578-1585. https://doi.org/10.1007/s00125-014-3272-2

(12) Murillo, S., Brugnara, L., Maduell, X., & Novials, A. (2020). Management of Hypoglycemia in Adults with Type 1 Diabetes in Real-Life Condition. *Annals of Nutrition and Metabolism*, *76*(4), 277-284.

(13) Singh, P., Jain, A., & Kaur, G. (2004). Impact of hypoglycemia and diabetes on CNS: correlation of mitochondrial oxidative stress with DNA damage. *Molecular and cellular biochemistry*, *260*(1), 153-159.

(14) Gogitidze Joy, N., Hedrington, M. S., Briscoe, V. J., Tate, D. B., Ertl, A. C., & Davis, S. N. (2010). Effects of acute hypoglycemia on inflammatory and pro-atherothrombotic biomarkers in individuals with type 1 diabetes and healthy individuals. *Diabetes care*, *33*(7), 1529-1535.

(15) Wang, J., Alexanian, A., Ying, R., Kizhakekuttu, T. J., Dharmashankar, K., Vasquez-Vivar, J., ... & Widlansky, M. E. (2012). Acute exposure to low glucose rapidly induces endothelial dysfunction and mitochondrial oxidative stress: role for AMP kinase. *Arteriosclerosis, thrombosis, and vascular biology*, *32*(3), 712-720.

(16) Charlton, A., Garzarella, J., Jandeleit-Dahm, K. A., & Jha, J. C. (2020). Oxidative stress and inflammation in renal and cardiovascular complications of diabetes. *Biology*, *10*(1), 18.

(17) Dos Santos, J. M., Tewari, S., & Mendes, R. H. (2019). The role of oxidative stress in the development of diabetes mellitus and its complications. *Journal of Diabetes Research*, *2019*.

(18) Ceriello, A., Novials, A., Ortega, E., La Sala, L., Pujadas, G., Testa, R., ... & Giugliano, D. (2012). Evidence that hyperglycemia after recovery from hypoglycemia worsens endothelial function and increases oxidative stress and inflammation in healthy control subjects and subjects with type 1 diabetes. *Diabetes*, *61*(11), 2993-2997.

(19) Colinet, V., & Lysy, P. A. (2022). Characterization of Post-Hypoglycemic Hyperglycemia in Children and Adolescents With Type 1 Diabetes : The EPHICA Study. *Frontiers in Endocrinology*, *13*. https://doi.org/10.3389/fendo.2022.887976

(20) Zhou, Z., Sun, B., Huang, S., Zhu, C., & Bian, M. (2020). Glycemic variability: adverse clinical outcomes and how to improve it?. *Cardiovascular diabetology*, *19*(1), 1-14.

(21) Pollé, O. G., Delfosse, A., Martin, M., Louis, J., Gies, I., Den Brinker, M., ... & DIATAG Working Group:. (2022). Glycemic Variability Patterns Strongly Correlate With Partial Remission Status in Children With Newly Diagnosed Type 1 Diabetes. *Diabetes care*, *45*(10), 2360-2368.

(22) Cole, T. J., & Lobstein, T. (2012). Extended international (IOTF) body mass index cut-offs for thinness, overweight and obesity. *Pediatric obesity*, *7*(4), 284-294.

(23) Watkins, W., & Taylor, M. (1993). CALCULATING AREAS OF IRREGULAR POLYGONS. *Problems, Resources, and Issues in Mathematics Undergraduate Studies*, *3*(4), 379-388

(24) Mortensen, H. B., Hougaard, P., Swift, P., Hansen, L., Holl, R. W., Hoey, H., ... & Åman, J. (2009). New definition for the partial remission period in children and adolescents with type 1 diabetes. *Diabetes care*, *32*(8), 1384-1390.

(25) Wentworth, J. M., Bediaga, N. G., Giles, L. C., Ehlers, M., Gitelman, S. E., Geyer, S., ... & Harrison, L. C. (2019). Beta cell function in type 1 diabetes determined from clinical and fasting biochemical variables. *Diabetologia*, *62*(1), 33-40.

(26) Simes, R. J. (1986). An improved Bonferroni procedure for multiple tests of significance. *Biometrika*, *73*(3), 751-754.

(27) Nakagawa, S., & Schielzeth, H. (2013). A general and simple method for obtaining R2 from generalized linear mixed-effects models. *Methods in ecology and evolution*, *4*(2), 133-142.

(28) Bates, D., Mächler, M., Bolker, B., & Walker, S. (2014). Fitting linear mixed-effects models using lme4. *arXiv preprint arXiv:1406.5823*.

(29) Kuznetsova, A., Brockhoff, P. B., & Christensen, R. H. (2017). lmerTest package: tests in linear mixed effects models. *Journal of statistical software*, *82*, 1-26.

(30) Stephenson, J. M., & Schernthaner, G. (1989). Dawn phenomenon and Somogyi effect in IDDM. *Diabetes Care*, *12*(4), 245-251.

(31) Rickels, M. R., Evans-Molina, C., Bahnson, H. T., Ylescupidez, A., Nadeau, K. J., Hao, W., ... & Greenbaum, C. J. (2020). High residual C-peptide likely contributes to glycemic control in type 1 diabetes. *The Journal of clinical investigation*, *130*(4), 1850-1862.

(32) Akirav, E., Kushner, J. A., & Herold, K. C. (2008). β-cell mass and type 1 diabetes: going, going, gone?. *Diabetes*, *57*(11), 2883-2888

(33) Hramiak, I. M., Dupre, J., & Finegood, D. T. (1993). Determinants of clinical remission in recent-onset IDDM. *Diabetes Care*, *16*(1), 125-132.

(34) Greenbaum, C. J., Mandrup-Poulsen, T., McGee, P. F., Battelino, T., Haastert, B., Ludvigsson, J., ... & Type 1 Diabetes Trial Net Research Group and the European C-Peptide Trial Study Group. (2008). Mixed-meal tolerance test versus glucagon stimulation test for the assessment of β-cell function in therapeutic trials in type 1 diabetes. *Diabetes care*, *31*(10), 1966-1971.

(35) Nalysnyk, L., Hernandez-Medina, M., & Krishnarajah, G. (2010). Glycaemic variability and complications in patients with diabetes mellitus: evidence from a systematic review of the literature. *Diabetes, Obesity and Metabolism*, *12*(4), 288-298.

(36) Nathan, D. M., Turgeon, H., & Regan, S. (2007). Relationship between glycated haemoglobin levels and mean glucose levels over time. *Diabetologia*, *50*(11), 2239-2244.

(37) Monnier, L., Colette, C., Wojtusciszyn, A., Dejager, S., Renard, E., Molinari, N., & Owens, D. R. (2017). Toward defining the threshold between low and high glucose variability in diabetes. *Diabetes care*, *40*(7), 832-838.

(38) Martyn-Nemeth, P., Quinn, L., Penckofer, S., Park, C., Hofer, V., & Burke, L. (2017). Fear of hypoglycemia: influence on glycemic variability and self-

management behavior in young adults with type 1 diabetes. *Journal of Diabetes and its Complications*, *31*(4), 735-741

Click here to access/download;Manuscript;Tables and figures legends.pdf

Tables and figures

Table 1. Cohort description and distribution of PHH parameters.

	Global	Remitters	Non-remitters	p-values
	**(*n*= 193)**	**(*n*= 108)**	**(*n*= 85)**	
**Phenotypic characteristics**				
**Age – years (at diagnosis)**	10.7 ± 3.41	11.3 ± 3.34	9.8 ± 3.34	0.05 †
**Sex – Male no (%)**	95 (50)	71 (66)	24 (30)	$7.3e^{-7}$ ‡
**Pubertal – no (%)**	98 (52)	64 (59)	34 (42)	0.02 ‡
**Parameters of glycemic homeostasis**				
**Clinical parameters**				
**Hb_{A1C} – %**	6.6 ± 1	6 ± 0.5	7.3 ± 0.9	$2.09\ e10^{-20}$ †
**$IDAA_{1C}$**	9 ± 1.6	7.9 ± 0.7	10.4 ± 1.2	$5.32\ e^{-34}$ †
**Continuous glucose monitoring metrics**				
**CV – %**	38.9 ± 8.6	36.1 ± 8.8	42.5 ± 6.5	$7.18e^{-8}$ †
**MODD – %**	47.9 ± 18.6	38.2 ± 13.5	60.6 ± 16.5	$2.01e^{-18}$ †
**Mean glycemia – mg/dL**	134.5 ± 30	118.5 ± 17.2	155.7 ± 30.3	$1.83e^{-17}$ †
**$TIR_{70\text{-}180}$ – %**	71.7 ± 15	79.4 ± 11.1	61.5 ± 13.4	$6.35e^{-18}$ †
**$TBR_{<70}$ – %**	9.5 ± 7.5	10.8 ± 8.4	7.6 ± 5.8	$4.60e^{-3}$ †
**$TAR_{>180}$ – %**	19.7 ± 16.2	10.8 ± 9	31.5 ± 16	$1.1e^{-18}$ †
**PHH parameters**				
**Frequency – no/day**	0.19 ± 0.20	0.15 ± 0.19	0.25 ± 0.2	$9.05e^{-5}$ ‖
**PHH_{AUC}**	32023 ± 30037	18895 ± 17387	48220 ± 34278	$6.51e^{-12}$ ‖
**PHH duration mean (minutes)**	155 ± 117	105 ± 75	217 ± 130	$3.38e^{-11}$ ‖
**PHH/hyperglycemia duration ratio – no**	0.04 ± 0.05	0.02 ± 0.02	0.07 ± 0.06	$1.43e^{-10}$ ‖

**PHH/hypoglycemia frequency ratio – no**	0.35 ± 1.06	0.28 ± 1.02	0.44 ± 1.12	$4.71e^{-7}$ ‖
**Circadian rhythm of PHH frequency – no**				
**Morning$_{5\text{-}10}$**	0.04 ± 0.07	0.04 ± 0.09	0.04 ± 0.06	1 ‖
**Day$_{10\text{-}4}$**	0.18 ± 0.27	0.10 ± 0.26	0.26 ± 0.32	$1.15e^{-4}$ ‖
**Evening$_{4\text{-}10}$**	0.11 ± 0.17	0.06 ± 0.10	0.17 ± 0.21	$1.92e^{-5}$ ‖
**Night$_{10\text{-}5}$**	0.03 ± 0.05	0.01 ± 0.02	0.04 ± 0.06	0.07 ‖

Plus-minus values are means ± SD. Percentages may not total 100 due to rounding. Glycemic homeostasis markers and PHH parameters were evaluated at +3, +6, +9, +12 months after diagnosis. Differences between remitters and non-remitters were considered as significant when p-value was under 0.05. † student t-test; ‡ Chi-square; ‖ Linear mixed models using Satterthwaite t-test. PHH, post-hypoglycemia hyperglycemia; HbA_{1C}, Glycated hemoglobin; $IDAA_{1C}$, Insulin Dose-Adjusted A1C = HbA_{1C} + 4 x insulin doses/day/kg; CV, coefficient of variation for glucose; MODD, mean of daily differences; $TIR_{70\text{-}180}$, Time in range (70-180 mg/dL); $TBR_{<70}$, Time below the range (<70 mg/dL); $GIIH_{<54}$, Grade 2 hypoglycemia (<54 mg/dL); $TAR_{>180}$, Time above the range (>180 mg/dL); Hyperglycemia$_{>250}$, Hyperglycemia above 250 mg/dL; PHH_{AUC}: area under the curve of PHH; PHH/Hyperglycemia duration ratio, ratio between the total duration of PHH and the total duration of hyperglycemia; PHH/Hypoglycemia frequency ratio, ratio between daily frequency of PHH and daily frequency of hypoglycemia; Morning$_{5\text{-}10,}$ morning time between 5 am and 10 am; Day$_{10\text{-}4,}$ daytime between 10 am and 4 pm, Evening$_{4\text{-}10,}$ evening time between 4 pm and 10 pm; Night$_{10\text{-}5,}$ nighttime between 10 pm and 5 am.

Table 2. Linear mixed-models determination coefficients between parameters of PHH and glucose homeostasis.

	PHH frequency		PHH_{AUC}		PHH duration mean		PHH/Hyperglycemia duration ratio		PHH/Hypoglycemia frequency ratio	
	R^2	p-values	R^2	p-values	R^2	p-values	R^2	p-values	R^2	p-values
CV	0.24	$9.4e^{-10}$	0.22	$5.82e^{-8}$	0.20	$2.9e^{-7}$	0.50	$2.2e^{-16}$	0.001	0.67
$IDAA_{1c}$	0.10	$2.3e^{-5}$	0.32	$4.04e^{-12}$	0.34	$1.01e^{-11}$	0.30	$4.97e^{-13}$	0.10	$1.54e^{-4}$
MODD	0.05	$1.2e^{-3}$	0.43	$2.2e^{-16}$	0.40	$3.6e^{-16}$	0.45	$2.2e^{-16}$	0.17	$4.47e^{-7}$
$Hypoglycemia_{<60}$ frequency	0.19	$1.5e^{-10}$	0.02	0.07	0.03	$3.7e^{-2}$	0.07	$2.4e^{-4}$	0.12	$7.88e^{-6}$
$TIT_{63\text{-}140}$	0.04	$4.4e^{-3}$	0.40	$2.2e^{-16}$	0.38	$2.2e^{-16}$	0.30	$2.2e^{-16}$	0.27	$1.60e^{-11}$
$TIR_{70\text{-}180}$	0.06	$9.3e^{-5}$	0.35	$9.32e^{-16}$	0.35	$6.69e^{-16}$	0.40	$2.2e^{-16}$	0.18	$3.74e^{-8}$
$TAR_{>180}$	0.01	0.06	0.42	$2.2e^{-16}$	0.41	$2.2e^{-16}$	0.28	$3.74e^{-15}$	0.24	$3.51e^{-10}$
$CPEP_{EST}$#	0.08	$6.3e^{-3}$	0.17	$2.68\ e^{-4}$	0.15	$6.6e^{-4}$	0.29	$9.56e^{-9}$	0.03	0.13

Marginal R-squared (coefficient of determination) were calculated using generalized linear mixed models. Results were considered as significant when p-value was under 0.05. Glycemic homeostasis markers and PHH parameters were evaluated at +3, +6, +9, +12 months after diagnosis. PHH, post-hypoglycemia hyperglycemia; Frequency, quantity of PHH per day; PHH_{AUC}, area under the curve of PHH; PHH/Hyperglycemia duration ratio, ratio between the total duration of PHH and the total duration of hyperglycemia; PHH/Hypoglycemia frequency ratio, ratio between daily frequency of PHH and daily frequency of hypoglycemia; $IDAA_{1C}$, Insulin Dose-Adjusted A1C = HbA1C + 4 x insulin doses/day/kg; CV, coefficient of variation for glucose; MODD, mean of daily differences; $TIR_{70\text{-}180}$, Time in range; $TIT_{63\text{-}140}$, Time in target; $TAR_{>180}$, Time above the range; $CPEP_{EST}$, estimation of c-peptide secretion calculated as described in Wentworth *et al*[25].

Figure 1 legend: Graphical representation of a continuous analysis of subcutaneous glucose (vertical axis) over a period of 9 hours (horizontal axis). The normoglycemia range is 60-160 mg/dL. In this example: end of hypoglycemia at 00:20 p.m., onset of PHH at 01:20 p.m., end of PHH at 07:45 p.m. Hyperglycemia occurred less than 2 hours after the end of hypoglycemia; PHH lasted for 6 hours and 25 minutes. The green area is the PHH AUC.

Figure 2 legend: Determination coefficient of PHH frequency (A-C), PHH_{AUC} (D-F) and PHH/Hyperglycemia duration ratio (G-I) according to glycemic homeostasis markers**.** Parameters of PHH parameters and glycemic homeostasis markers were obtained at +3, +6, +9, and +12 months after the diagnosis. Marginal R-squared (coefficient of determination) were calculated using generalized linear mixed models. The vertical *red dashed lines* represent specific thresholds of glucose homeostasis parameters (CV=36%, time in 70-180 mg/dL range=70% and $IDAA_{1C}$ score=9). The horizontal *red dashed lines* represent specific status-related thresholds of PHH parameters (PHH frequency=0.14, PHH_{AUC}=26567 and PHH/Hyperglycemia duration ratio=0.02). Panels **A-C** represent the regression results with 95% CI bands (*shaded zone*) between PHH frequency and CV (A), TIR (B) and $IDAA_{1C}$ (C). Panels **D-F** represent the regression results with 95% CI bands (*shaded zone*) between PHH_{AUC} and CV (D), time in 70-180 mg/dL range (E) and $IDAA_{1C}$ (F). Panels **G-I** represent the regression results with 95% CI bands (*shaded zone*) between PHH frequency and CV (G), time in 70-180 mg/dL range (H) and $IDAA_{1C}$ (I). Correlation coefficients (R) are shown according to the PHH frequency (**A-C**), PHHAUC (**D-F**) and PHH/Hyperglycemia duration ratio (**G-I**); Abbreviations: PHH frequency (number/day), PHH frequency; Average PHH area under the curve (AUC), PHH_{AUC}; PHH Hyperglycemia ratio, PHH/Hyperglycemia duration ratio; CV (%), coefficient of variation for glucose; Time in 70-180 mg/dL range, Time in range; IDAA1C score, Insulin Dose-Adjusted A1C = HbA_{1C} + 4 x insulin doses/day/kg. The level of significance of the correlations is represented after the *regression coefficient* as follows: nonsignificant (*ns*), p<0.05 (*), p<0.01 (**), p<0.001 (***).

Figure 3 legend: Multidimensional representation of PHH parameters according to the three target patient groups identified using hierarchical clustering. PHH parameters and glycemic homeostasis markers were obtained at +3, +6, +9 and +12 months after the diagnosis. Three-dimensional representation of PHH_{AUC}, frequency of hypoglycemia and PHH Hypo ratio. Each color of the dots is specific to a target group (green=Group 1, red=Group 2, blue=Group 3).

Figure 4 legend: Distribution of PHH parameters according to the glucotypes [21]. PHH parameters and glycemic homeostasis markers were obtained at +3, +6, +9 and +12 months after the diagnosis. The glucotypes were determined as described by our

group [21]. **(A)** Boxplots of PHH frequency according to glucotypes (group 1-4). **(B)** Boxplot of PHH/hyperglycemia duration ratio according to glucotypes (group 1-4). Size of the dots corresponds to the PHH duration mean. Color of the dots represents levels of PHH_{AUC}, using continuous colored gradient scale (*green-purple*) with PHH_{AUC}=26567 in *white*. Abbreviations: PHH frequency, quantity of PHH per day; PHH AUC, area under the curve of PHH; Mean HPH duration, average duration of a PHH; Log (PHH ratio), logarithm function of ratio between the total duration of PHH and the total duration of hyperglycemia.

 Click here to access/download;Figure;Figures.pdf

Figure 1

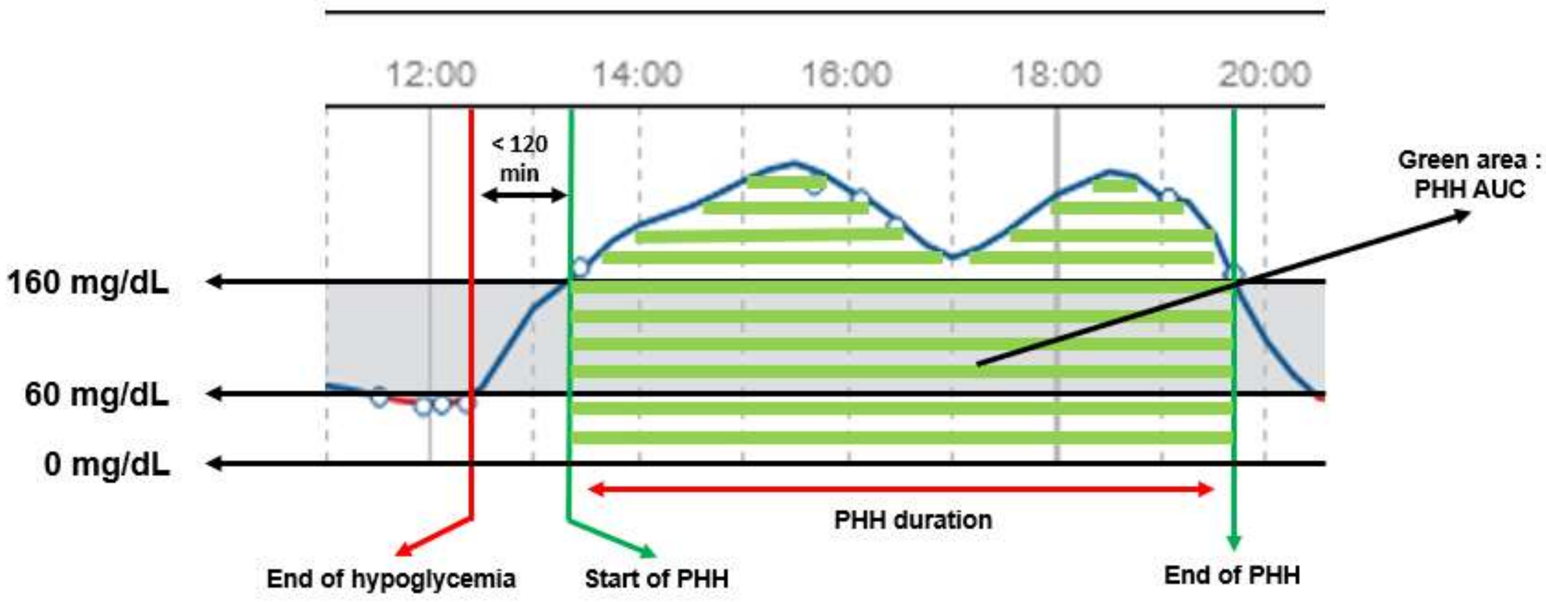

Figure 2

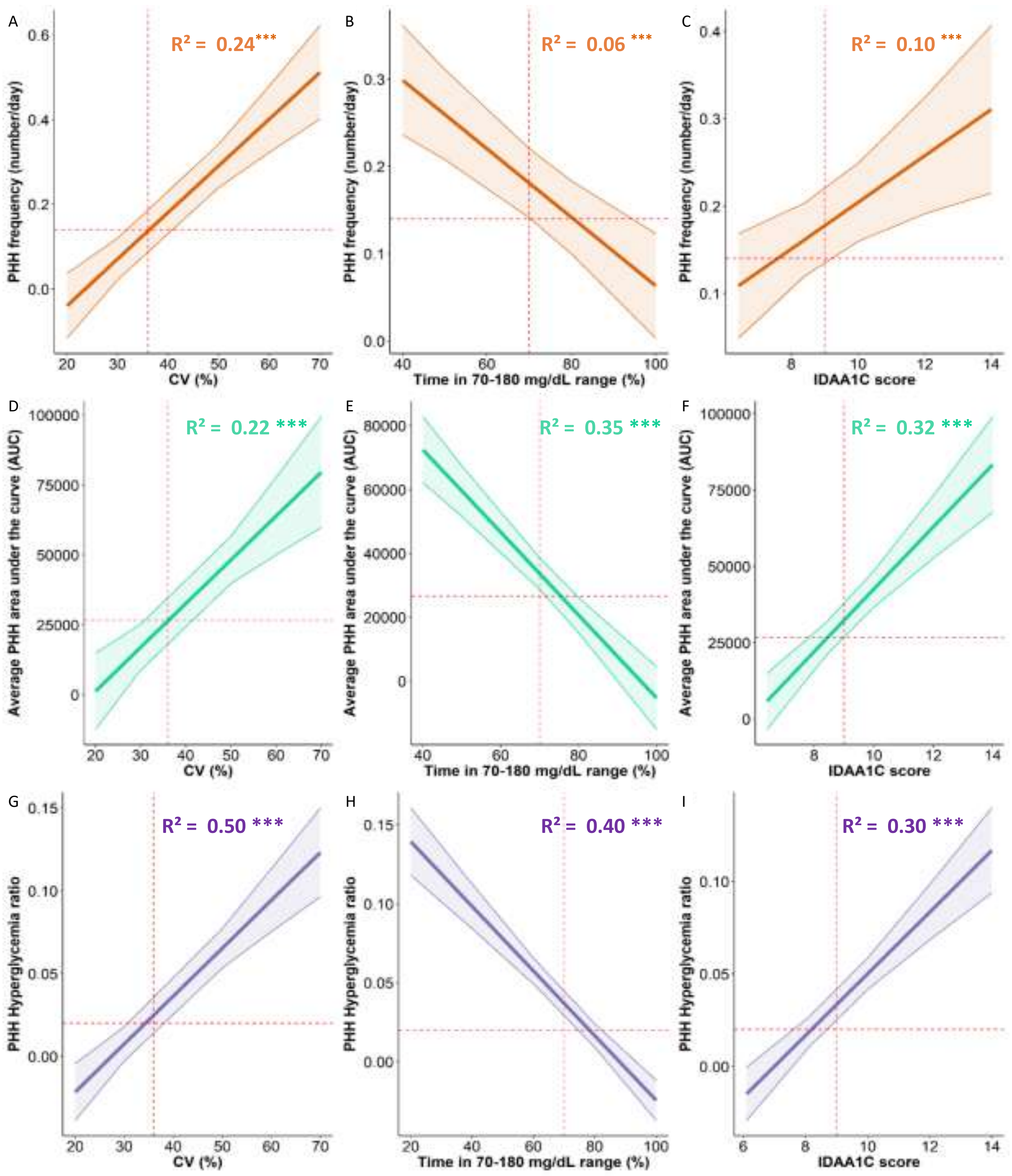

A
R² = 0.24***
PHH frequency (number/day)
CV (%)
B
R² = 0.06 ***
PHH frequency (number/day)
Time in 70-180 mg/dL range (%)
C
R² = 0.10 ***
PHH frequency (number/day)
IDAA1C score
D
R² = 0.22 ***
Average PHH area under the curve (AUC)
CV (%)
E
R² = 0.35 ***
Average PHH area under the curve (AUC)
Time in 70-180 mg/dL range (%)
F
R² = 0.32 ***
Average PHH area under the curve (AUC)
IDAA1C score
G
R² = 0.50 ***
PHH Hyperglycemia ratio
CV (%)
H
R² = 0.40 ***
PHH Hyperglycemia ratio
Time in 70-180 mg/dL range (%)
I
R² = 0.30 ***
PHH Hyperglycemia ratio
IDAA1C score

Figure 3

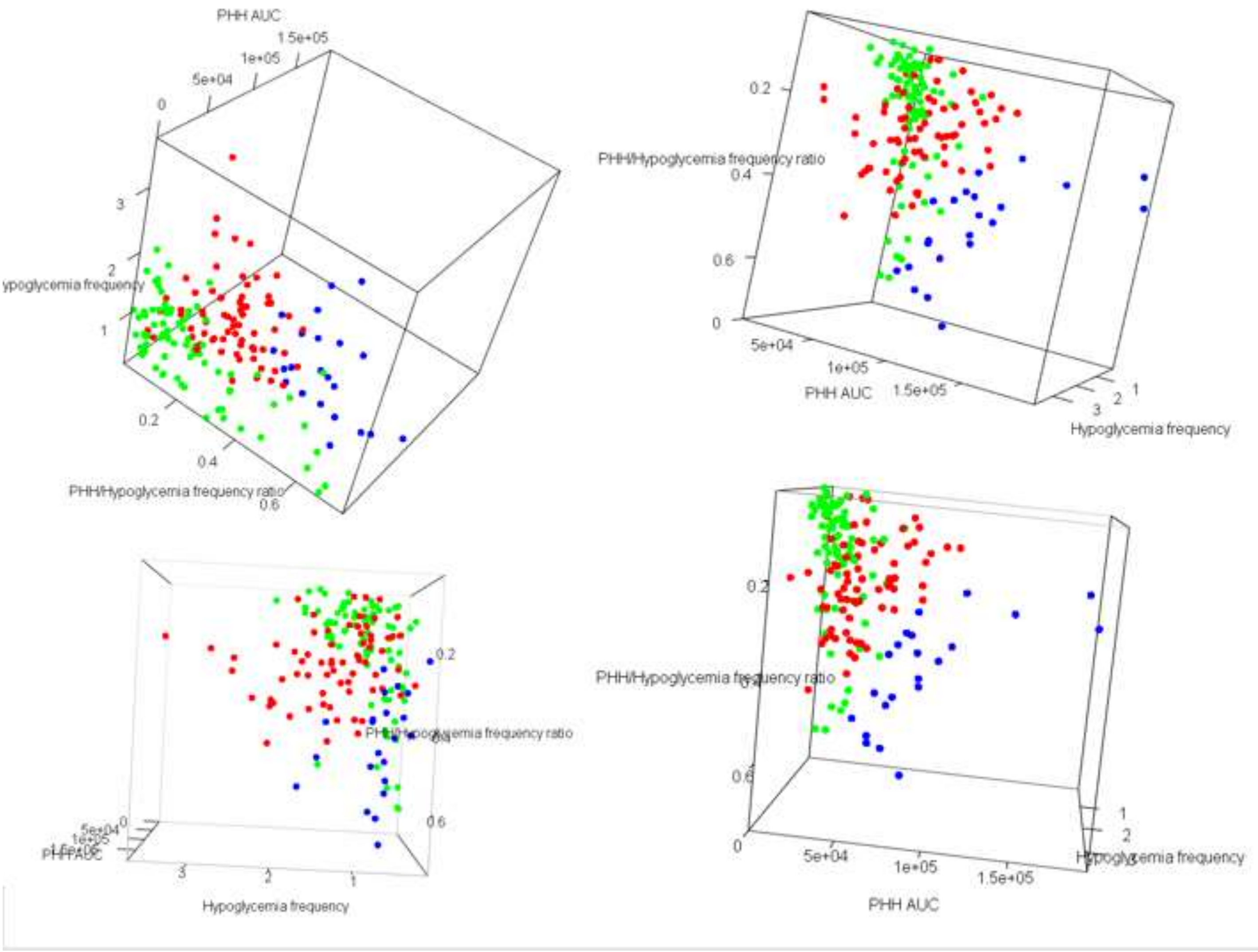
PHH AUC
1.5e+05
1e+05
5e+04
0
3
2
ypoglycemia frequency
1
0.2
0.4
PHH/Hypoglycemia frequency ratio
0.6
0.2
PHH/Hypoglycemia frequency ratio
0.4
0.6
0
5e+04
1e+05
PHH AUC
1.5e+05
3
2
1
Hypoglycemia frequency
0.2
PHH/Hypoglycemia frequency ratio
0.6
3
2
1
Hypoglycemia frequency
0.2
PHH/Hypoglycemia frequency ratio
0.6
0
5e+04
1e+05
1.5e+05
PHH AUC
1
2
Hypoglycemia frequency

Figure 4

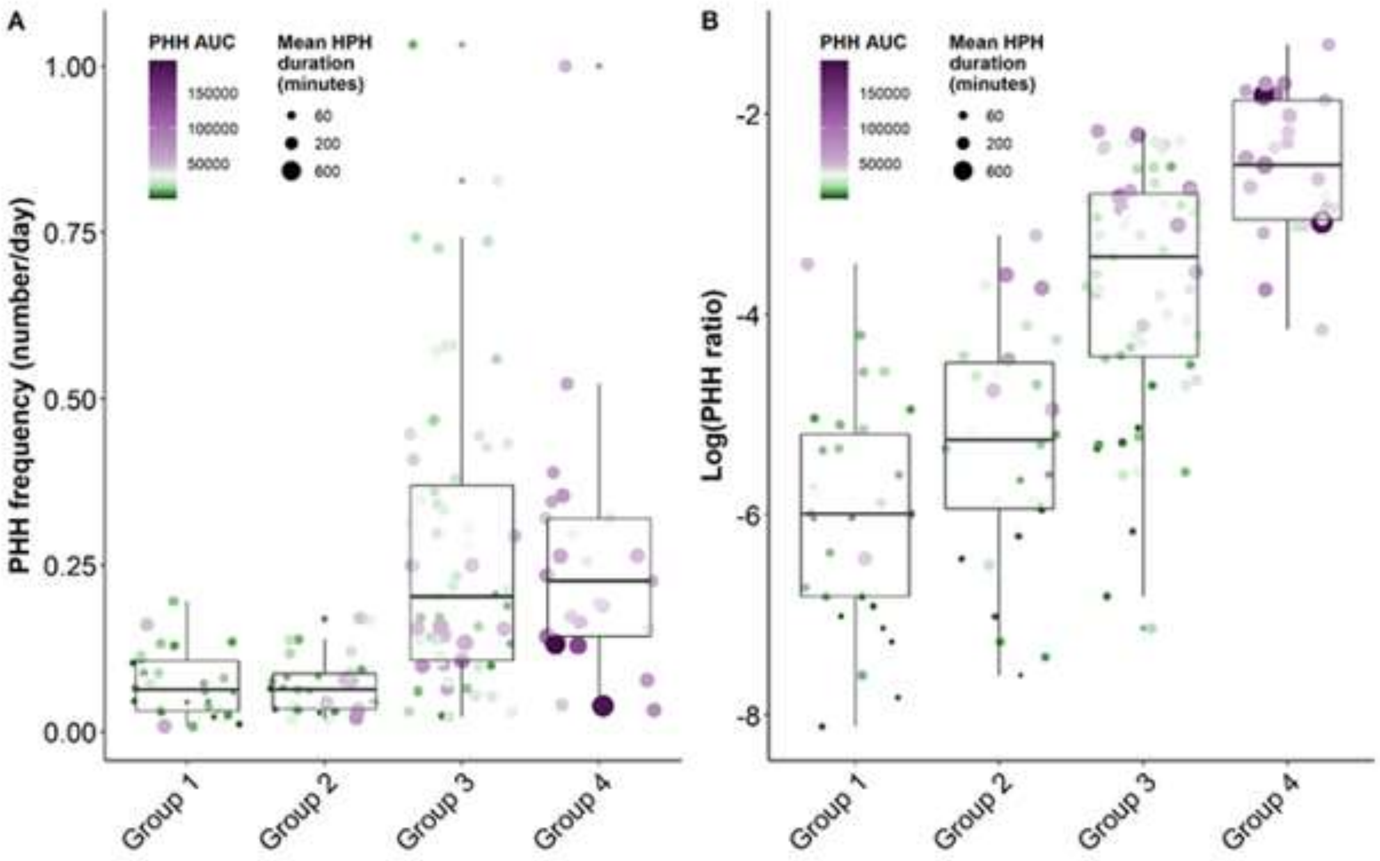
A
PHH AUC
150000
100000
50000
Mean HPH duration (minutes)
60
200
600
PHH frequency (number/day)
1.00
0.75
0.50
0.25
0.00
Group 1
Group 2
Group 3
Group 4
B
Log(PHH ratio)
-2
-4
-6
-8
