# Supplementary material for: Post-Hypoglycemic hyperglycemia are highly relevant markers for stratification of glycemic variability and partial remission status of pediatric patients with new-onset type 1 diabetes
Source: PLoS One. 2023 Nov 30;18(11):e0294982. doi: 10.1371/journal.pone.0294982 (PMC10688654; doi:10.1371/journal.pone.0294982)
Supplement: Attachment — Submitted filename: Reponse to reviewers.pdf [file pone.0294982.s003.pdf]

Abstract

There are several grammatical errors that authors need to correct. Moreover, there are abbreviations that need to be defined at first mention. Under the results, authors need to quote the estimated that indicate these PHH parameters can discriminate between remitters and non-remitters.

Methodology

Under continuous glucose monitoring, authors indicate varied devices being used for this purpose but fail to indicate how these were standardized for the purpose of this study. This section requires further proof reading (see attached reviewer pdf). Authors do not justify the sample size used in the study.

Results

Authors should re-check the total number of outpatient visits in the first paragraph. There are sections under frequency of PHH which are better suited for methodology or discussion (see attached reviewer pdf). Authors base conclusions on R^2 values that are minimal with statistical significance and must discuss with this in mind. Authors must thus be cautious of their conclusions on these estimates.

Discussion

The first paragraph under the discussion is more of a literature for background than the beginning of discussion which usually has the major findings from the results. The inclusion criteria of experience with COVID-19 need further information as the frequency and how recent are likely to influence responses.

Conclusion

The Conclusion should specifically speak to aims of the study followed by implications of these findings.

Minor Comments

See attached reviewer’s comments

Reviewer's Responses to Questions

Comments to the Author

1. Is the manuscript technically sound, and do the data support the conclusions?

The manuscript must describe a technically sound piece of scientific research with data that supports the conclusions. Experiments must have been conducted rigorously, with appropriate controls, replication, and sample sizes. The conclusions must be drawn appropriately based on the data presented.

Reviewer #1: No

Reviewer #2: Yes

---

2. Has the statistical analysis been performed appropriately and rigorously?

Reviewer #1: Yes

Reviewer #2: Yes

---

3. Have the authors made all data underlying the findings in their manuscript fully available?

The PLOS Data policy requires authors to make all data underlying the findings described in their manuscript fully available without restriction, with rare exception (please refer to the Data Availability Statement in the manuscript PDF file). The data should be provided as part of the manuscript or its supporting information or deposited to a public repository. For example, in addition to summary statistics, the data points behind means, medians and variance measures should be available. If there are restrictions on publicly sharing data—e.g. participant privacy or use of data from a third party—those must be specified.

Reviewer #1: Yes

Reviewer #2: Yes

---

4. Is the manuscript presented in an intelligible fashion and written in standard English?

PLOS ONE does not copyedit accepted manuscripts, so the language in submitted

articles must be clear, correct, and unambiguous. Any typographical or grammatical errors should be corrected at revision, so please note any specific errors here.

Reviewer #1: No

Reviewer #2: Yes

---

5. Review Comments to the Author

Please use the space provided to explain your answers to the questions above. You may also include additional comments for the author, including concerns about dual publication, research ethics, or publication ethics. (Please upload your review as an attachment if it exceeds 20,000 characters)

Reviewer

#1: Abstract
The abstract should be restructured with the results section revised to include means percentages and standard deviations.

We thank the reviewer for the useful comments. Statistical values were added to the abstract as requested. Grammatical changes were also made in the manuscript.

What was the design of the study?

The present GLUREDIA study included data from the DIATAG multicentric and prospective study. Clarifications on this topic were added in the revised version of the manuscript in page 7.

The authors should indicate how the glycated hemoglobin was estimated?

Glycated hemoglobin was measured from venous blood samples using high performance liquid chromatography. The reference cut-offs are those described by Tosoh (reference range 4–6% [20–42 mmol/mol]) (Tosoh G8; Tosoh, Tokyo, Japan). This precision was added in the revised version of the manuscript on page 7.

What is the definition of glucotypes and how did the authors come by the types?

Glucotypes correspond to daily glucose patterns. These were identified by our team during the 1rst year of type 1 diabetes in pediatric patients in the context of the DIATAG study (Pollé et al., Diabetes Care 2022). Patients with new-onset type 1 diabetes

distributed across these four glucotypes, progressively, from 3 months onwards. The glucotypes were identified using hierarchical clustering that included a combination of CGM metrics and clinical parameters (i.e., $IDAA_{1C}$, HbA_{1C}, IDD). This precision was already presented in the previous version of the manuscript (page 9).

The limitation should be added to the main text of the discussion.

Limitations were described as requested.

The length of the conclusion should also be reduced.

Conclusion length was reduced by more than 40% in the present manuscript (i.e., from 179 words to 103 words).

Reviewer #2: General Comment

This manuscript is a well thought of experiment that evaluates whether parameters of post-hypoglycemic hyperglycemia (PHH) correlated with glycemic homeostasis during the first year after type 1 diabetes onset and tries to use this to distinguish pediatric patients undergoing partial remission or not.

We thank the reviewer for this kind comment.

However, there are some specific comments that need attention.

Abstract;
There are several grammatical errors that authors need to correct. Moreover, there are abbreviations that need to be defined at first mention. Under the results, authors need to quote the estimates that indicate these PHH parameters can discriminate between remitters and non-remitters.

We have added the statistical data requested by Reviewer #1 in the abstract. Grammatical changes were also made in the manuscript. Also, the conclusion was revised as follows: “PHH parameters are new minimal-invasive markers to discriminate remitters from non-remitters and to assess glycemic homeostasis during the first year of type 1 diabetes that allow patient-specific therapeutic management”.

Methodology
Under continuous glucose monitoring, authors indicate varied devices being used for this purpose but fail to indicate how these were standardized for the purpose of this study. This section requires further proof reading (see attached reviewer pdf).

As mentioned in our previous article that used a similar dataset, most patients were under Freestyle Libre CGM (i.e., >90%), and no sensor-specific pattern was observed within the principal component analysis [1]. This information was already mentioned in the first version of the manuscript (i.e., limitations on page 20). We nonetheless added additional precisions (i.e., PCA results from DIATAG study).

Authors do not justify the sample size used in the study.

The sample size was calculated at the initiation of DIATAG study by Pr. Annie Robert using PR occurrence as the major outcome. In recent studies, the partial remission period (i.e., IDAA1C < 9) has a mean of 9 months with a range from 2 to 24 months. Dividing 22 months by 6 provided an estimate of the standard deviation σ=3.7 months. With a sample size of 82 patients randomized in 2 balanced groups of 41 patients, the phase III clinical trial will have a power of 95% to detect an increase from 9 to 12 months in remission period, at the 0.05 statistical significance level.

Results

Authors should re-check the total number of outpatient visits in the first paragraph. There are sections under frequency of PHH which are better suited for methodology or discussion (see attached reviewer pdf).

We reviewed the total number of patients included in every analysis and corrected numbers accordingly in the manuscript. There was 244 outpatients visits with 52 that did not fully fulfilled inclusion criteria. Final analysis was performed on 192 visits from 66 patients.

Authors base conclusions on R2 values that are minimal with statistical significance and must discuss with this in mind. Authors must thus be cautious of their conclusions on these estimates.

We thank the reviewer for this comment. We describe the strength of the correlation using the guide that Evans (1996). The strength is defined like: very weak (R^2 < 0.03), weak (R^2 : [0.03 – 0.15]), moderate (R^2 : [0.16 – 0.35]), strong (R^2 : [0.36 – 0.62]) and very strong (R^2 > 0.62) of

Discussion

The first paragraph under the discussion is more of a literature for background than the beginning of discussion which usually has the major findings from the results.

We thank the reviewer for this remark. We introduced our discussion with this paragraph to contextualize our research and remind principal hurdles in current literature. Thereby, we would prefer keeping the latter as it is now. However, if requested by the Editor or the Reviewer, we would certainly remove it. Major findings are summarized in the second paragraph of the discussion.

The inclusion criteria of experience with COVID-19 need further information as the frequency and how recent are likely to influence responses.

Two of our patients discontinued the whole study during COVID-19 pandemic.
None of our patients included in DIATAG study spontaneously reported a COVID-19 infection during data collection. However, there was no systematic PCR testing at each visit.
Finally, recent studies highlighted that during COVID pandemic, global glucose levels remained stable in youth with type 1 diabetes, thereby supporting the reliability of our study despite having accurate COVID-19 detection [2,3]. We think that adding such information may not improve the current manuscript.

Conclusion
The Conclusion should specifically speak to aims of the study followed by implications of these findings.

Conclusion was rewritten accordingly.

---

6. PLOS authors have the option to publish the peer review history of their article (what does this mean?). If published, this will include your full peer review and any attached files.

If you choose "no", your identity will remain anonymous, but your review may still be made public.

Do you want your identity to be public for this peer review? For information about this choice, including consent withdrawal, please see our Privacy Policy.

Reviewer #1: **Yes:** RICHARD KD EPHRAIM

Reviewer #2: No

References

1. Pollé, O. G., Delfosse, A., Martin, M., Louis, J., Gies, I., Den Brinker, M., ... & DIATAG Working Group:. (2022). Glycemic variability patterns strongly correlate

with partial remission status in children with newly diagnosed type 1 diabetes. *Diabetes care*, *45*(10), 2360-2368.

2. Choudhary A, Adhikari S, White PC. Impact of the COVID-19 pandemic on management of children and adolescents with Type 1 diabetes. BMC Pediatr. 2022;22(1):1–7.
3. Danne T, Lanzinger S, De Bock M, Rhodes ET, Alonso GT, Barat P, et al. A Worldwide Perspective on COVID-19 and Diabetes Management in 22,820 Children from the SWEET Project: Diabetic Ketoacidosis Rates Increase and Glycemic Control Is Maintained. Diabetes Technol Ther. 2021;23(9):632– 41
