# Supplementary material for: Post-Hypoglycemic hyperglycemia are highly relevant markers for stratification of glycemic variability and partial remission status of pediatric patients with new-onset type 1 diabetes
Source: PLoS One. 2023 Nov 30;18(11):e0294982. doi: 10.1371/journal.pone.0294982 (PMC10688654; doi:10.1371/journal.pone.0294982)
Supplement: Attachment — Submitted filename: PONE_D_22_33441_SND.docx [file pone.0294982.s002.docx]

**Abstract**;

There are a number of grammatical errors that authors need to correct. Moreover, there are abbreviations that need to be defined at first mention. Under the results, authors need to quote the estimated that indicate these PHH parameters are able to discriminate between remitters and non-remitters.

**Methodology**

Under continuous glucose monitoring, authors indicate varied devices being used for this purpose but fail to indicate how these were standardized for the purpose of this study. This section requires further proof reading (see attached reviewer pdf). Authors do not justify the sample size used in the study

**Results**

Authors should re-check the total number of outpatient visits in the first paragraph. There are sections under frequency of PHH which are better suited for methodology or discussion (see attached reviewer pdf). Authors base conclusions on R^2^ values that are minimal with statistical significance and must discuss with this in mind. Authors must thus be cautious of their conclusions on these estimates.

***Discussion***

The first paragraph under the discussion is more of a literature for background than the beginning of discussion which usually has the major findings from the results. The inclusion criteria of experience with COVID-19 need further information as the frequency and how recent are likely to influence responses

**Conclusion**

The Conclusion should specifically speak to aims of the study followed by implications of these findings.

**Minor Comments**

See attached reviewer’s comments
