# Supplementary material for: Post-Hypoglycemic hyperglycemia are highly relevant markers for stratification of glycemic variability and partial remission status of pediatric patients with new-onset type 1 diabetes
Source: PLoS One. 2023 Nov 30;18(11):e0294982. doi: 10.1371/journal.pone.0294982 (PMC10688654; doi:10.1371/journal.pone.0294982)
Supplement: Attachment — Submitted filename: Additional Editor Comments.docx [file pone.0294982.s004.docx]

**Additional Editor Comments:**

Authors has responded adequately to most of the concern raised by the reviewers, however, some few outstanding concern,

We thank the editor for their constructive comments.

1. the revised manuscript were not numbered, and this make it difficult to relate the responses to the changes, authors should number the pages.

The pages are now numbered.

2. Authors should not assume everybody has read their previous study, the current manuscript should be able to make meaning to it readers without recourse to their earlier paper, authors should factor this in their write up.

We have added an explanatory paragraph regarding our recent published research in the GLUCOTYPE paragraph (pages 9 - 10 )

3. still first-time usage of most acronyms are not defined.

We have specified each acronym used in our article (see "Manuscript").

4. table 1 what do authors mean by global ??? do they mean total??

In our Table 1, Global means our entire cohort. For ease of understanding, we have replaced the word "**Global**" with the word "**Total**".
