# Supplementary material for: Post-Hypoglycemic hyperglycemia are highly relevant markers for stratification of glycemic variability and partial remission status of pediatric patients with new-onset type 1 diabetes
Source: PLoS One. 2023 Nov 30;18(11):e0294982. doi: 10.1371/journal.pone.0294982 (PMC10688654; doi:10.1371/journal.pone.0294982)
Supplement: Attachment — Submitted filename: Reviewer.docx [file pone.0294982.s006.docx]

**Reviewer #3: Remarks to the authors
Manuscript Number: PONE-D-22-33441R2**

**Summary of the research**

This study is interestingly addressing the partial remission occurred in the first year after the onset of type 1 Diabetes in pediatric patients. The fluctuations in blood glucose levels have unpleasant outcomes, and greatly affect the quality of life for the kids and their parents. Thus, I think the prediction of the glycemic variability using less invasive approach would contribute to highlight the best practices of therapeutic managements.
comments.

**Title**

The title is descriptive, concise, and interesting, but it would be more accurate if the partial remission was addressed :

The word "partial" was added in the title to clarify the notion that we are referring to the partial remission.

**Abstract**
The abstract is simple and specific, and well structured.
The "CGM" abbreviation should be defined at the first mention in the abstract. manuscript (i.e. CGM, continuous glucose monitoring) :

Abbreviation was added to the revised version of the manuscript.

**Introduction**
The authors start with a general overview of Type 1 diabetes, and the concerns over the incidences of glycemic variability in the pediatric patients. The research question was introduced through reviewing the most recent literature. The authors also stated the drawbacks of other markers used to address the hypoglycemic and hyperglycemic events (i.e. β-cell function and Insulin-Dose Adjusted A1C).
Finally, the key characteristics and the aim of the study were outlined.

**Methodology**
The authors stated the criteria of participants' enrollment in the study, and they also have considered the ethical basis of their work. All the methods, data collection and procedures were clearly described.

In the "partial remission" section, why did you only depend on IDAA1C in the classification of remitters and non-remitters according to reference #24?

We thank the reviewer for the comment. Indeed, while IDAA_1C_ score has some drawbacks, this is the most common and accepted definition of partial remission in patients with type 1 diabetes (including ISPAD ^1^) and was validated in major study cohorts ^2-4^. Therefore, we chose to use the latter definition to classify remitters from non-remitters.

1- Besser, R. E., Bell, K. J., Couper, J. J., Ziegler, A. G., Wherrett, D. K., Knip, M., ... & Haller, M. J. (2022). ISPAD Clinical Practice Consensus Guidelines 2022: Stages of type 1 diabetes in children and adolescents. *Pediatric diabetes*, *23*(8), 1175-1187.
2- Max Andersen, M. L. C., Hougaard, P., Pörksen, S., Nielsen, L. B., Fredheim, S., Svensson, J., ... & Mortensen, H. B. (2014). Partial remission definition: validation based on the insulin dose‐adjusted HbA1c (IDAA1C) in 129 Danish children with new‐onset type 1 diabetes. *Pediatric diabetes*, *15*(7), 469-476.
3- Nagl, K., Hermann, J. M., Plamper, M., Schröder, C., Dost, A., Kordonouri, O., ... & Holl, R. W. (2017). Factors contributing to partial remission in type 1 diabetes: analysis based on the insulin dose‐adjusted HbA1c in 3657 children and adolescents from Germany and Austria. *Pediatric Diabetes*, *18*(6), 428-434.
4- Chiavaroli, V., Derraik, J. G., Jalaludin, M. Y., Albert, B. B., Ramkumar, S., Cutfield, W. S., ... & Jefferies, C. A. (2019). Partial remission in type 1 diabetes and associated factors: Analysis based on the insulin dose‐adjusted hemoglobin A1c in children and adolescents from a regional diabetes center, Auckland, New Zealand. *Pediatric diabetes*, *20*(7), 892-900.

A recently published research (2022) have pointed out the limitation of this criteria, which was published in 2009 study. So, please refer to "Nwosu BU. Partial Clinical Remission of Type 1 Diabetes: The Need for an Integrated Functional Definition Based on Insulin-Dose Adjusted A1c and Insulin Sensitivity Score. Front Endocrinol (Lausanne). 2022 May 3;13:884219. doi: 10.3389/fendo.2022.884219. PMID: 35592786; PMCID: PMC9110823"

As mentioned before, all definitions of partial remission have drawbacks. Nonetheless, IDAA_1C_ score is the most representative of these as it includes two parameters used in clinical routine (i.e., HbA1C and insulin daily dose). We do not understand why adding this reference may improve the manuscript.

However, if the reviewer feel it may improve the manuscript, we may add it.
**Results**
The findings of research questions were clearly presented in the results section. However, I have noticed that these results were more presented in tables and visualized as figures according to the participant's status classification (i.e. remitters versus non-remitters), rather than the four glucotypes. Is there a specific explanation for that?

We thank the reviewer for this comment. Glucotypes were described by our team and are still not validated in other studies. In the present study, we wanted to explore whether these parameters differed amongst (and distinguished) glucotypes, which they actually did. We chose to present most results according to the remission status (based on IDAA1C) as it is the most commonly used definition of PR. However, **Figure 4**  and the last paragraph of the Result section describe PHH parameters according to glucotypes.

**Discussion and Conclusion**
The authors did highlight the findings of the study and compare the results between the main glucotypes under investigation. They have described the strengths of the study as well as the limitations. However, they did not introduce suggestions to overcome these limitations for PHH markers as a potential markers of PR.

PHH, either isolated or in combination, enables us to accurately distinguish the patient's remission status. By opting for a global rather than isolated approach, we showed that these parameters provide a more precise view of the patient's clinical situation (remitters vs. no-remitters). A considerable advantage is that they require no biological sampling, making their assessment accessible at any time.

These parameters also constitute an interesting new clinical tool, going beyond simple description, as they give additional information on patient’s hypoglycemic status that may foster a better clinical management, and thus improve the management of their diabetes. The ability to target patients requiring therapeutic education to better manage their hypoglycemia makes these parameters a major asset in the overall approach to diabetes care.

Finally, Do the authors suggest any future studies that need to be carried out?

We believe it would be useful to evaluate these clinical parameters in independent and larger cohorts of patients with type 1 diabetes (adult patients, various ethnic backgrounds, other countries, long-term diabetes).

**References**
The References listed are relevant and mostly are recently published.
Modifications have been made, all in APA format.

**Figures**
I think the figures need improvements in terms of font size of the labels, resolution, and the thickness of lines and ticks.

All the figures have been reworked. They have been submitted in a Powerpoint file in TIFF format.

**Tables**
Modifications have been made; the number of decimals is identical for each parameter except in special cases.

**Reviewer #4: 1. Summary of the research**
The main research question is clear and well-defined, that is to asses the correlation, if any, between PHH parameters and glucose homeostasis in newly diagnosed T1D pediatric patients and differentiate remitters from nonremitters using least invasive technique, which is another noble goal.
Except for some undefined abbreviations, the introduction is clear, concise and informative and gives a good background to the audience about the current subject.
As for the study design I found it ambiguous in many points as in the true number of recruiters and then the number of CGM metrics which represents the sample size, Plus referring to a previous study for the reader to visit and extract the details. Or even mentioning a certain detail that does not match the previous study which referred to as the mother study. This was misleading and exhausting indeed.

All abbreviations were reviewed.

For patients, a flowchart was created to better visualize the evolution of the number of patients.
The results are described in detail using clear language and is presented by more than one form (tables and graphs). Figure 1 is really fascinating as it unravels what words could not clarify in the manuscript. However, although I am not a statistics expert, sometimes the deduction does not match the data presented by the figure.

All statistics analysis were performed by an expert statistician (Manon Martin) and verified by a second biostatistician (Laurent Gatto). Linear mixed models were used to take into account multiple measurements from the same patient. Finally, the whole manuscript was reviewed by both Manon Martin and Laurent Gatto.

**An edited form of the manuscript is attached to view all the review point and comments but below are some examples of which.**
2. Examples and evidence
Major issues:
a. In the abstract, methods section, first line: what does GLUREDIA abbreviate? If it has a full term, please mention it first and if not, I suggest defining it shortly before mentioning it or just say: "in the current study, named GLUREDIA, ..." or " In this study, called GLUREDIA ...."This is because mentioning it in the abstract preceded by the article "the" gives the impression that it is a well-defined famous-in-the-field abbreviation, which is not true and hence misleading to the audience

GLUREDIA: **Glu**cagon **Re**sponse to Hypoglycemia in children and adolescents with new-onset type 1 **dia**betes.

The changes have been added to the revised manuscript.
b. The same for " EPHICA " in the introduction fourth paragraph, 3rd line and "DIATAG" in the study design section first paragraph, 1st line.

EPHICA: **E**valuation of **P**osthypoglycemic **H**yperglycemia **i**n **C**hildren and **A**dolescents with diabetes.

DIATAG: **DIA**betes **TAG**ging

The changes have been added to the manuscript.
c. The repeated referring to the authors' previous works with hiding some important details to force the reader to go back to these previous studies is uncomfortable, giving the impression it is not a standalone study, besides revealing some controversies with those studies like in the study design the authors mentioned that the current study is subsidiary from the "DIATAG". It is also mentioned that the age upper limit is 17 years. Returning to the DIATAG study in reference 21, the upper limit for age was 18 not 17. In the current study, is the upper limit for age 18 like in the DIATAG and this is a typo? or there is a reason for excluding those aged above 17. please specify it. So, please mention the exact age range in this section.

It is indeed a typo and patients were aged between 6 months and 18 years old. We did not exclude any specific patients for age criteria. Information was modified accordingly in the revised manuscript.

d. Upon visiting the registered clinical trial (NCT04007809) to which this study belongs the true number of recruited participants was 98, What were the reasons for excluding these 27 participants? Please mention the exact number of participants clearly in this section.

A flowchart was added to the revised manuscript to better visualize the total number of the patients and the reasons for patient’s exclusion.

e. The exclusion criteria also were found at the registered clinical trial (NCT04007809) not in reference 21 as mentioned in the manuscripts. So, from the abovementioned (c, d & e) if the authors had decided to include only 71 participant from the 98, by selecting them randomly before the study began, and age range up to 17 only not 18, with no convincing reason, I suggest mentioning all the study details, conditions, criteria in the methodology section, to present the current study as an independent one without referring to previous works or clinical trials.

A flowchart was added to the revised manuscript to better visualize the total number of the patients and the reasons for patient’s exclusion.

f. In the introduction fourth paragraph, 3rd line defining the PHH as hypoglycemia followed by hyperglycemia does not sound good. because English-wise the term refers to hyperglycemia that is described by being post hypoglycemic, i.e happens after hypoglycemia, (unless you mean to describe the whole phenomena, which will not be compatible with what I understood from Fig 1 )
the suggested rephrased definition is found in the attached document.

We thank the reviewer for his remark. The sentence was replaced by “PHH was defined as hyperglycemia (i.e., glycemia >160 mg/dL), that is preceded, within a 2-hour period, by hypoglycemia (i.e., glycemia <60 mg/dL) (Fig. 1).” in the revised manuscript.

g. In the results section under "Clinical and anthropometric characteristics of the GLUREDIA cohort" the manipulation of the numbers is not clearly understood. In other words, there were no justifications for the decrease in number in the first step:
- How does quarterly visits in a year duration for 71 patients count for 244? i.e: 71*4= 284, not 244, so you should mention that there were 40 missing visits, before mentioning the exclusion of 52 visits from the 244 that gives 192 , the supposed (192) visits that corresponds to 66 patients (provided not every patient from the 66 attended all the 4 visits), was then after under the title "PHH is frequent during the first year……..etc." seen as (194) CGM metrics. Then, it appears as (193) glucose records under the title " Partial remission is associated with fewer and lower PHH occurrence ". Again, in table 1 n=194. So, what is the right number 194, 192 or 193?
- I think this is a critical issue that affect all the numbers in calculations and statistics.

A flowchart was added to the revised manuscript to better visualize the total number of the patients and the reasons for patient’s exclusion.

h. Where would a reader find all the data which the authors have referred to as " fully available without restriction, in any part of the manuscript, or in another public repository or as a supplementary material?

Our patients' CGMs will not be available on any platform. In order to have access to our CGM data, it is necessary to contact the corresponding author and establish a data transfer contract in accordance with the ethical and legal rules.

i. In the results which hyperglycemia is meant in (PHH/Hyperglycemia duration ratio)?

Hyperglycemia duration refer to the total time spent in hyperglycemia (in minutes).
